# Partnering with Palliative Care: A Case Report of Severe Pain in Critical Limb Ischemia Treated Successfully with a Continuous Popliteal Nerve Catheter

**DOI:** 10.1155/2020/1054521

**Published:** 2020-04-06

**Authors:** Ryan S. D'Souza, Stephanie Shen, Frederick Ojukwu, Halena M. Gazelka, Bridget P. Pulos

**Affiliations:** ^1^Department of Anesthesiology and Perioperative Medicine, Mayo Clinic, 200 1^st^ St SW, Rochester, MN 55904, USA; ^2^Department of Anesthesiology and Perioperative Medicine, Division of Pain Medicine, Mayo Clinic, 200 1^st^ St SW, Rochester, MN 55904, USA; ^3^Center for Palliative Medicine, Mayo Clinic, 200 1^st^ St SW, Rochester, MN 55904, USA

## Abstract

**Background:**

Critical limb ischemia (CLI) is limb pain occurring at rest or impending limb loss as a result of lack of blood flow to the affected extremity. CLI pain is challenging to control despite multimodal pharmacologic analgesia and surgical intervention. We described the successful use of a continuous local anesthetic infusion via a popliteal nerve catheter to control severe refractory ischemic lower limb pain in a patient who failed surgical intervention and performed a brief narrative literature review on regional anesthesia for ischemic pain. *Case Presentation*. A 74-year-old female with acute myelogenous leukemia presented with CLI after experiencing left popliteal artery occlusion. Palliative medicine service was consulted for pain management in the setting of escalating narcotic dose requirements. She experienced a complicated hospital course with several failed attempts at surgical revascularization due to arterial rethrombosis. In accordance with the patient's goals of care, a continuous popliteal nerve catheter was placed, despite the high risk nature of an intervention in an immunocompromised patient with thrombocytopenia (platelet count of 30,000 platelets/microliter) and ongoing therapeutic anticoagulation. The patient experienced immediate relief while transitioning to comfort care.

**Conclusion:**

This is the first report of successful analgesia for CLI via a continuous popliteal catheter in a patient with rethrombosis and failed surgical revascularization. Based on our collaborative experience, we recommend the development of partnerships between the acute pain service and palliative care service to facilitate the early evaluation and decision to utilize regional anesthesia for treatment of CLI.

## 1. Background

Critical limb ischemia (CLI) is a source of significant pain and distress that afflicts nearly 1 million patients in the United States [1]. CLI is defined as limb pain occurring at rest or impending limb loss as a result of lack of blood flow to the affected extremity [2]. The pathophysiology of ischemic pain is multifactorial and poorly understood but is thought to be due to inadequate oxygen causing release of chemical mediators including histamine, potassium ions, hydrogen ions, and bradykinin, which subsequently lead to nociceptor sensitization and primary hyperalgesia [3]. It is challenging to control ischemic pain despite implementation of multimodal pharmacologic analgesia.

Peripheral nerve blockade is extensively used for perioperative pain management. However, controversy exists regarding the efficacy of regional anesthesia techniques in treating ischemic pain. Current evidence is limited to observational studies and expert opinion regarding axial and peripheral nerve blocks, primarily in patients with acute compartment syndrome. We describe a case of CLI from acute arterial thrombosis, refractory to pharmacologic analgesia and inoperable by surgical intervention, which was successfully controlled with a continuous local anesthetic infusion via a popliteal nerve catheter. We also perform a narrative review on regional anesthesia for ischemic pain.

## 2. Case Presentation

A 74-year-old female presented to a local hospital with severe left lower extremity pain and oral thrush. In the emergency department, laboratory data revealed anemia, thrombocytopenia of 10,000 platelets/microliter, and elevated white blood count of 235,500 leukocytes/microliter with 97% blasts concerning for leukemia. Bone marrow biopsy confirmed a new diagnosis of acute myelogenous leukemia (AML). She was subsequently transferred to a major tertiary referral center for management.

On further evaluation, she continued to have worsening left lower extremity pain with physical exam notable for cyanosis of all five toes, erythema to the midshin, and decreased sensation to touch but intact posterior tibial and dorsalis pedis pulses detected using a Doppler device. Bilateral lower extremity ultrasound revealed a left popliteal artery occlusion at the level of the popliteal fossa and acute bilateral deep vein thrombosis (DVTs). The patient was started on a heparin infusion for acute DVT and given nonopioid adjuvants (scheduled oral acetaminophen, scheduled oral gabapentin, lidocaine patch, and ketamine injection as needed) as well as oral and intravenous opioids to control her pain. Despite escalation of pain regimen up to 200 oral morphine equivalents (OME), she continued to experience 10/10 pain at rest and was unable to sleep at night. Palliative care service was consulted for further management.

**Figure 1:**
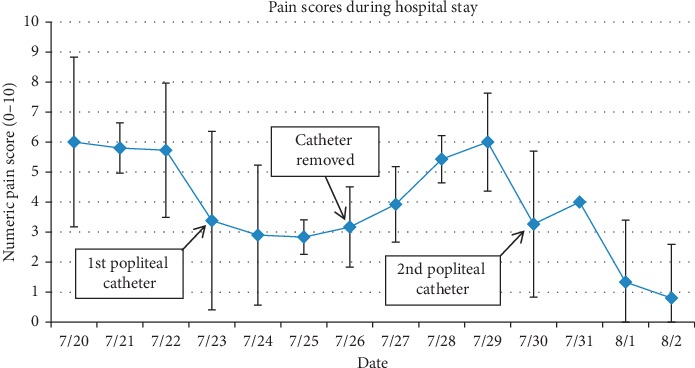
Pain scores during hospital stay. The line graph displays mean numeric pain scale scores for each hospital day, ranging from 0 (no pain) to 10 (worst possible pain). Standard deviation bars are displayed for each day.

**Figure 2:**
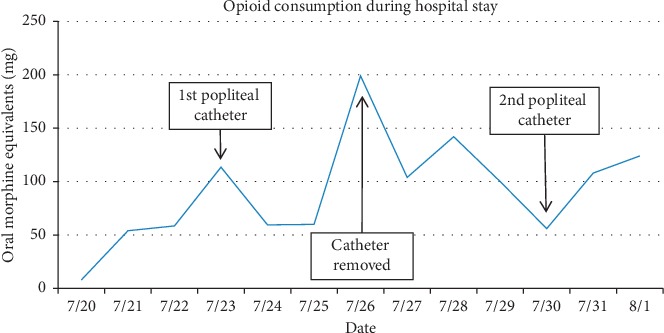
Opioid consumption during hospital stay. The line graph displays oral morphine equivalents (in mg) consumed for each hospital day. A peak of 200 OME was observed on 7/26 when the catheter was removed.

tGiven her worsening pain, a regional anesthetic technique was considered. Due to her critical thrombocytopenia and continuous heparin infusion, she was not a candidate for neuraxial anesthesia. Consideration was given to the patient's multiple risk factors for peripheral nerve block including bleeding, infection due to severe immunocompromised state, and potential for masking a developing compartment syndrome or a worsening of her CLI. After an extensive discussion with the patient and her family regarding the high risk nature of an invasive procedure, the decision was made to proceed with a trial of continuous popliteal nerve catheter for the patient's refractory pain. A left popliteal nerve catheter was placed by the anesthesia acute pain service under ultrasound guidance using classic landmarks with an initial bolus dose of 30 mL of 0.25% bupivacaine followed by an infusion of 0.1% bupivacaine at 10 mL per hour. The patient reported near immediate pain relief with pain score decreasing to 0/10. She was able to sleep through the night, transitioned off intravenous narcotics, and only required minimal oral narcotics for breakthrough pain (less than 60 mg OME per day). In Figure 1, we display average patient-reported pain scores for each hospital day, utilizing a numeric pain scale ranging from 0 (no pain at all) to 10 (worst pain possible). In Figure 2, we display the total 24-hour OME consumed by the patient for each hospital day. We manually converted the reported opioid dosage for each hospital day to its respective OME using the Mayo Opioid Converter Online Tool (https://kmt-prod-opioidui.mayo.edu/#/converter; accessed June 2019).

**Figure 3:**
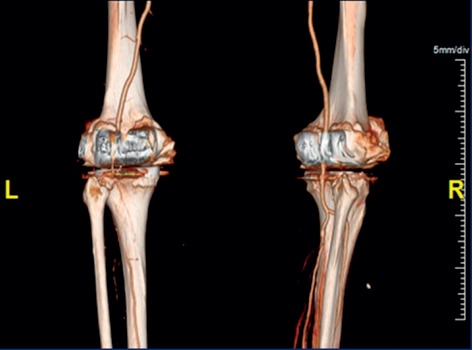
Computed tomography (CT) angiography. This CT image revealed left popliteal, left deep femoral, and left anterior tibial artery occlusion.

Subsequently, the patient had a complicated hospital course. A follow-up CT scan showed increased clot burden to the level of the aorta, and she underwent an attempt at surgical revascularization of the affected limb with thromboembolectomy (Figure 3). For the surgery, a left femoral artery cutdown was performed and the thrombus was found within the distal aorta, left common iliac artery, left external iliac artery, profunda femoris, and left popliteal artery. A transverse left common femoral arteriotomy was performed with retrograde thromboembolectomy of the left common iliac artery and the external iliac artery. A second transverse arteriotomy for anterograde thromboembolectomy from the left popliteal artery was also performed. A completion angiogram demonstrated patency of the aforementioned occluded vessels.

Unfortunately, the left popliteal artery acutely rethrombosed in the postanesthesia care unit, resulting in an emergent return to the operating room for a second attempt at revascularization. During this surgery, there was thrombosis in the left distal superficial femoral artery and the left proximal popliteal artery. Balloon angioplasty and stenting of the left distal superficial femoral artery and left proximal popliteal artery were performed, and a completion angiography demonstrated patency and inline flow down to the left posterior tibial artery. Although the second surgery initially appeared to be successful, the patient again experienced rethrombosis of the popliteal artery the next morning. Following discussion with the patient, her family, and the multidisciplinary care team, further surgical intervention was felt to be unlikely to provide significant benefit and not to be in accordance with her goals of care. The peripheral nerve catheter had been removed at the time of the first surgery, and the patient's severe ischemic leg pain returned. A new ultrasound-guided left popliteal nerve catheter was placed with near immediate pain relief. She was then transitioned to comfort care with the catheter in place and ultimately died five days later, likely due to complications of AML. Consent was obtained from the patient's family to publish this report.

## 3. Discussion

We presented the case of a 74-year-old female with critical limb ischemia from popliteal artery occlusion who was treated palliatively with continuous popliteal nerve blockade because she could not receive the standard course of curative surgical revascularization. We demonstrated that regional anesthesia may be an effective modality to treat severe pain associated with CLI, despite failed surgical attempts at revascularization. While the mechanism of pain in CLI is unclear, it is probable that the pain experienced by our patient was a component of ischemia, local tissue damage, and inflammation. Chemical mediators released from ischemic tissue may sensitize sensory afferent fibers, resulting in primary hyperalgesia. We speculate that the continuous popliteal nerve infusion produced pain relief by attenuating the conduction of noxious stimuli via primary afferent nerve fibers.

In addition to a poorly understood mechanism, there is a paucity of data on the use of interventional regional techniques to manage ischemic pain in CLI. The MEDLINE, Google Scholar, and Embase databases were searched through June 8, 2019, using a highly sensitive text word search strategy to find any reports, observational studies, and randomized controlled trials (RCT) describing use of any peripheral nerve blockade with intermittent or continuous local anesthetic to treat any type of ischemic pain. Serial searches included the terms “critical limb ischemia,” “peripheral arterial disease,” “ischemic pain,” “Buerger's disease,” “Raynaud's phenomenon,” “chronic limb ischemia,” “regional anesthesia,” “local anesthetic,” “analgesia,” and “block” independently and in combination using Boolean operators. Studies involving cadavers, volunteers, or neuraxial anesthesia were excluded. Specific outcomes addressed included quality of analgesia and complications.

Our search yielded 617 total studies after duplicates were removed and 20 full-text articles that were assessed for eligibility. No RCTs or other Level I or II evidence-based trials were identified. Ten articles (three observational studies [4–6] and seven case reports/series [7–13] met inclusion criteria for review, which are displayed in Table 1. While no prospective observational studies regarding peripheral nerve catheters in this population have been reported, all three included retrospective studies [4–6] and five of seven case reports/series [7–9, 12, 13] demonstrated improved analgesia for ischemic pain after implementation of regional anesthesia. Location of ischemia was variable including digital ischemia in six studies [7–9, 11–13], critical limb ischemia of the lower extremities in four studies [5, 6, 10, 11], and upper limb ischemia in one study [4]. Type of ischemic pathology was also variable comprising gangrene (atherosclerotic, thromboembolic, diabetic dry, and post-traumatic), scleroderma, Buerger's disease, and complex regional pain syndrome (CRPS).

Tureli and colleagues [6] documented successful use of popliteal nerve blockade to provide adequate intraoperative analgesia for 30 patients with CLI undergoing urgent endovascular treatment, with 87% of patients reporting visual analog scale (VAS) scores of 0 (no pain) and 13% of patients reporting VAS scores from 1 to 3 (mild to annoying pain). However, all patients in the series were revascularized and long-term pain control in patients with rethrombosis was not studied [6]. Similarly, another retrospective observational study comprising 25 patients [5] with lower limb CLI demonstrated that combined sciatic and femoral nerve blockade was associated with significant reduction in ischemic rest pain (mean VAS score 3.7) permitting angioplasty without neuraxial anesthesia or general anesthesia. Pertaining to upper extremity limb ischemia, only one observational study [4] was reported and comprised 20 patients with varying disease including gangrene (atherosclerotic, thromboembolic, diabetic dry, and post-traumatic), Raynaud's disease, and 1 CRPS patient. Results from this study were promising with a significant reduction in pain (preblock VAS score 7 and post-block VAS score 4.25), mean duration of analgesia of 7 hours, and 100% pain relief by the 12^th^ week in 18 of 20 patients. However, results may be confounded by the regional anesthetic injectate containing adjunct ketamine. Most included studies generally reported improved quality of life and avoidance of opioid escalation after regional anesthesia blockade.

Another potential benefit from regional anesthesia is increase in blood flow to the affected tissue, possibly due to sympathetic blockade, which may be advantageous to the medical improvement in limb ischemia [11, 14]. It is well known that gradual ischemia of nerves and tissues activates the sympathetic system, accelerating a vicious cycle of pain-vasospasm-ischemia-gangrene [4]. The dysfunctionality of the sympathetic nervous system with subsequent loss of local autoregulation may be a predictor of early amputation [15]. Thus, timely implementation of regional anesthesia may achieve a favorable sympathectomy with localized improvement in blood flow, and subsequently resolution of ischemia. This was evident in several included studies that reported resolution of gangreneous ulcers, avoidance of amputation, and resolution of other concerning physical exam findings (e.g., cyanotic toes and cold extremities) [4, 8, 9]. It is possible that our patient experienced improved pain control due to increased circulation to her left foot via sympathetic blockade.

Finally, complications were rare in included studies reflecting the overall safety of regional anesthesia for ischemic pain. Only one observational study [4] investigating stellate ganglion blocks reported transient Horner's syndrome in 12 patients, light-headedness in 16 patients, hoarseness in six patients not requiring any intervention, bradycardia in two patients responsive to low-dose intravenous glycopyrrolate, and hematoma in one patient which resolved in 12 hours.

The literature has more extensively described management of ischemic pain in compartment syndrome. This occurs when pressure within a defined compartmental space increases past a critical threshold subsequently decreasing perfusion pressure to that compartment and is an emergency requiring immediate surgical intervention [16]. Use of regional anesthesia, including neuraxial blockade and peripheral nerve blockade, has been described for acute compartment syndrome [17–20]. Despite concerns of masking pain that may be secondary to worsening compartment syndrome, most reports propose that ischemic pain is not masked or completely blocked by regional anesthesia, with the key to diagnosis being worsening pain in the setting of effective regional anesthesia [11, 21]. When regional anesthesia is used in patients at risk of compartment syndrome, all members of the care team must be educated on the signs and symptoms of compartment syndrome and worsening ischemia.

Another strategy for analgesia would be opioid escalation in our patient although we were concerned about associated side effects of nausea and vomiting, sedation, hallucination, respiratory depression, and ultimately decreased quality of life from these side effects. Furthermore, the high prevalence of renal insufficiency [22] and cognitive dysfunction [23, 24] in patients with CLI makes it challenging to administer high-dose opioids. The goal of care in this case was to improve quality of life and specifically the ability to communicate with family members. Thus, as displayed by improved pain scores in Figure 1 and reduced opioid consumption in Figure 2, regional anesthesia offered the best possibility of achieving this goal. Epidural blockade is also an effective analgesic modality although we were reluctant to offer this option in the setting of ongoing anticoagulation treatment and thrombocytopenia.

While effective pain control is crucial, the literature emphasizes other major goals of treating CLI including reducing ischemia, healing or preventing ischemic ulcer, preventing limb loss, and ultimately improving patient physical function and quality of life. Most studies focus on limb salvage as the primary outcome or include invasive surgical interventions. However, many patients with CLI are ultimately referred to palliative care for pain management services as they may be approaching end of life due to nonreconstructable CLI, such as in our case report. In fact, the Inter-Society Consensus for the Management of Peripheral Arterial Disease (TASC II) states that the management of CLI is usually palliative [25]. Moreover, patients may be unfit for surgery, while others may have persistent distal ischemia with pain even in the presence of a functioning revascularization [26].

To the best of our knowledge, this is the first report of successful analgesia for CLI via a continuous popliteal catheter in a patient with rethrombosis and failed surgical revascularization. Our plan for symptom management was made in collaboration with the patient, her family, and her multidisciplinary care team including palliative medicine, anesthesia acute pain, oncology, and vascular surgery teams. This partnership was essential to determine the optimal strategy given the patient's changing clinical course and evolving goals of care.

In conclusion, pharmacologic and surgical management remain the first-line therapy for ischemic limb pain, but in refractory cases, regional anesthesia may be effective for pain control. Based on our collaborative experience, we recommend the development of partnerships between the acute pain service and palliative care service to facilitate the early evaluation and decision to utilize regional anesthesia for treatment of CLI. The application of this technique may even be considered in an out-of-hospital palliative care setting. Future larger-scale prospective clinical trials should compare the efficacy of regional anesthetic techniques with traditional therapy in alleviating CLI pain. As spinal cord stimulation has recently been described for successful CLI treatment [26], regional anesthetic techniques should also be compared with this rapidly developing therapy.

## Figures and Tables

**Table 1:** Summary of findings from included studies.

Study	Disease/sample size	Type of regional blockade	Quality of analgesia	Procedural complications
Observational studies				

Kulkarni et al. 2010^a^	PVD of UE (20 pts)	Multiple interval single-shot stellate ganglion blocks (injectate contained ketamine also)	Preblock VAS of 7 decreased to 4.25 postblock; mean duration of analgesia 7 h; 100% pain relief at 12^th^ week in 18/20 pts; complete healing of gangreneous fingers in 17/19 pts	Horner's (12 pts), hoarseness (6 pts), hematoma (1 pt), bradycardia (2 pts), dizziness (16 pts)

Marcus et al. 2006	CLI in LE (25 pts)	Single-shot sciatic and femoral nerve block	All pts experienced reduction in ischemic rest pain permitting angioplasty without neuraxial anesthesia or GA; mean VAS 3.7 (scale 0–10)	None

Tureli et al. 2018	CLI in LE (30 pts)	Single-shot popliteal sciatic nerve block	VAS scores were 0 (no pain) in 87% of pts and 1–3 (mild to annoying pain) in 13% of pts	None

Case reports/series				

Belsky et al. 2015	Blue toe syndrome from thromboembolus (1 pt)	Single-shot digital block	Significant alleviation of ischemic pain	None

Greengrass et al. 2003	Digital ischemia in scleroderma (1 pt)	Continuous ambulatory axillary nerve block	Significant alleviation of ischemic pain, 6-months following removal of catheter, no recurrence of pain or ischemic ulcers was observed	None

Han et al. 2008	Digital ischemia in scleroderma (1 pt)	Continuous thoracic sympathetic ganglion block	VAS scores improved from a peak of 6/10 to 1/10; healing of medically refractory digital gangrene	None

Hashimoto et al. 2011	CLI in LE (2 pts with 1 having Buerger's disease)	Continuous popliteal sciatic nerve block which was changed to intermittent patient-controlled bolus	No pain alleviation with continuous infusion in both cases; pain alleviation noted with patient-controlled intermittent bolus in 1 case	None

Kucera and Boezaart 2014	Tight cast placement on right LE (1 pt); ischemia in right 4^th^/5^th^ fingers (1 pt)	Single-shot sciatic and femoral nerve block; continuous C7 paravertebral block	No relief of ischemic pain in both cases despite dense motor and sensory blockade	None

Saddler and Crosse 1988	Digital ischemia in Buerger's disease	Continuous ambulatory median nerve block	Significant alleviation of ischemic pain	None

Soberón et al. 2014	Digital ischemia of the 4^th^/5^th^ fingers (1 pt)	Continuous supraclavicular nerve block (became dislodged), followed by single-shot axillary block	Modest relief of ischemic pain	None

Some included studies added another adjunct in the injectate or utilized liposomal bupivacaine; ^a^Kulkarni et al included patients with gangrene (atherosclerotic, thromboembolic, diabetic dry, and post-traumatic), Raynaud's disease, and 1 CRPS patient and categorized them as having ischemic PVD. LE = lower extremity; UE = upper extremity; pts = patients; VAS = visual analog scale; PVD = peripheral vascular disease; h = hours; GA = general anesthesia.

## Abbreviations

CLI: Critical limb ischemia
AML: Acute myelogenous leukemia
DVT: Deep vein thrombosis
OME: Oral morphine equivalent
TASC II: Inter-Society Consensus for the Management of Peripheral Arterial Disease.
